# Glucose transporter-1 (GLUT-1): a potential marker of prognosis in rectal carcinoma?

**DOI:** 10.1038/sj.bjc.6601202

**Published:** 2003-08-26

**Authors:** R Cooper, S Sarioğlu, S Sökmen, M Füzün, A Küpelioğlu, H Valentine, I B Görken, R Airley, C West

**Affiliations:** 1 Department of Radiation Oncology, Dokuz Eylül University Medical School, Inciraltu, Izmir 35340, Turkey; 2 Department of General Surgery, Dokuz Eylül University Medical School, Izmir 35340, Turkey; 3 Department of Pathology, Dokuz Eylül University Medical School, Izmir 35340, Turkey; 4 Tumour Metabolism and Therapeutics Group, School of Pharmacy and Chemistry, John Moores University, Liverpool L3 3AF, UK; 5 Academic Department of Radiation Oncology, Christie Hospital, Manchester M20 4BX, UK

**Keywords:** GLUT-1, rectal cancer, hypoxia

## Abstract

The aim of the study is to evaluate the pattern and level of expression of glucose transporter-1 (GLUT-1) in rectal carcinoma in relation to outcome as a potential surrogate marker of tumour hypoxia. Formalin-fixed tumour sections from 43 patients with rectal carcinoma, who had undergone radical resection with curative intent, were immunohistochemically stained for GLUT-1. A mean of three sections per tumour (range 1–12) were examined. Each section was semiquantitatively scored; 0, no staining; 1, <10%; 2, 10–50%; 3, >50% and a score given for the whole section, the superficial (luminal) and deep (mural) part of the tumour. Staining was seen in 70% of tumours. Increased staining was noted adjacent to necrosis and ulceration. A diffuse and patchy pattern of staining, with and without colocalisation to necrosis was seen. Patients with high GLUT-1-expressing tumours (score 3 *vs* 0–2) had a significantly poorer overall survival (*P*=0.041), which was associated with poorer metastasis-free survival with no difference in local control. No significant correlation was seen with other prognostic factors. There was a strong correlation between the score for the superficial and deep parts of the tumour (*r*=0.81), but a significant relationship with outcome was only found in the deep part (*P*=0.003 *vs P*=0.46). In conclusion, increased GLUT-1 expression in rectal tumours was an adverse prognostic factor and is worth further evaluation as a predictive marker of response to therapy.

The presence of hypoxia in tumours is known to lead to resistance to radiotherapy and chemotherapy and is associated with a more aggressive phenotype with an increased propensity for metastases (Hockel *et al*, 1996; Brown *et al*, 1998; Dachs *et al*, 1998). This latter finding is thought to be related to the increased expression of a number of proteins acting through the HIF-1 pathway, which allows tumour cells to survive the harsh tumour microenviroment (Maxwell *et al*, 1997; Semenza, 2000). The glucose transporter-1 (Glut-1) is one of the proteins upregulated in hypoxic conditions.

Tumours show increased uptake of glucose compared to normal tissue (Isslbacher, 1972), a response mediated by a number of facilitative glucose transporters located in the cell membrane (Zhang *et al*, 1999). Glut-1 is one of this family of facilitative glucose transporters. It is expressed variably in normal tissue (Mueckler *et al*, 1985) and is responsible for the passive transport of glucose across the cell membrane (Chen *et al*, 2001). The function and expression of Glut-1 is regulated by a number of physiological and pathophysiological conditions (Zhang *et al*, 1999). In the tumour microenvironment, hypoxia results in increased transcription of the Glut-1 gene, mediated through HIF-1, and reduction in oxidative phosphorylation, in the absence of hypoxia, leads to increased stabilisation of the Glut-1 mRNA (Behrooz *et al*, 1997). Glucose transporter-1 is overexpressed in several different tumour types (Younes *et al*, 1996). In addition, increased expression has been shown to correlate with a poor prognosis in a variety of tumours (Younes *et al*, 1997; Haber *et al*, 1998; Airley *et al*, 2001; Cantuaria *et al*, 2001). More recently, Glut-1 expression has been shown to correlate with the level of tumour hypoxia in carcinoma of the cervix measured using either Eppendorf needle electrodes (Airley *et al*, 2001) or pimonidazole staining (Airley *et al*, 2003). Therefore, the level of Glut-1 expression might be a suitable surrogate or intrinsic marker of hypoxia, which could be measured simply and inexpensively as part of the routine histological assessment of tumours.

Little is known about the oxygenation of rectal tumours; however, it is likely that hypoxia plays a significant part in determining outcome (Haustermans *et al*, 2000; Wendling *et al*, 1984). Furthermore, determination of the level of hypoxia might be important for selecting appropriate preoperative radiotherapy or chemoradiotherapy regimes or determining which patients are more likely to develop distant metastases and therefore require systemic therapy. In this study, we report for the first time the level and pattern of expression of Glut-1, as a potential endogenous marker of hypoxia, in rectal carcinoma and relate the level of expression to outcome.

## PATIENTS AND METHODS

### Patients

The study was retrospective. Formalin-fixed tumour sections from 43 consecutive patients (29 male, 14 female) with rectal carcinoma who had undergone curative intent treatment at a single institution were analysed for GLUT-1 protein expression. For each patient, sections from all available tissue blocks were taken (median 2; range 1–12). The median age was 56 years (range 21–80). All patients underwent radical surgery with a curative intent. Only one patient underwent an R1 resection (microscopic residual disease), the remaining 42 underwent an R0 resection (no residual disease). Pathological tumour stages are summarised in Table 1

**Table 1:** Summary of patient and tumour characteristics

**Characteristic**	
Age (years)	
Median	57
Range	21–80

T stage	
T2	9
T3	29
T4	5

Nodal status	
Negative	27
Positive	16

Tumour size (pathological)	
Median (cm)	5
Range (cm)	2–11
<5 cm	19
⩾5 cm	24

Grade	
1	10
2	24
3	3
Not classified	6

. Adjuvant therapy was either preoperative radiotherapy/chemoradiotherapy (*n*=6), postoperative radiotherapy/chemoradiotherapy (*n*=18) or postoperative chemotherapy alone (*n*=3). Pre- and postoperative radiotherapy/chemoradiotherapy was administered in an outpatient setting. Patients undergoing radiotherapy were treated with 6 MV or ^60^Co gamma radiation. A four-field box technique was used extending from the junction of L4/L5 to the bottom of the obturator foramina and 1 cm beyond the lateral pelvic side walls. The field extended from the most posterior aspect of the sacrum to the posterior part of the pubic bone. The prescribed dose, specified to an appropriate isodose envelope, was 45 Gy in 25 fractions (1.8 Gy per fraction) given once daily 5 days per week. Concurrent chemotherapy was administered as a continuous infusion of 225 mg/m^−2^ 5-fluorouracil. Three patients received adjuvant chemotherapy consisting of 5-fluorouracil and folinic acid using the de Gramont regimen. The median follow-up for all patients was 46 months (range 8–102 months) and 49 months (range 30–102 months) for surviving patients.

### Immunohistochemistry

Formalin-fixed, paraffin-embedded tumour sections (5 *μ*m thick) were placed on poly-L-lysine-coated slides. Sections were dewaxed in xylene and rehydrated using a series of ethanol solutions of increasing dilution. Staining was carried out using the Envision Plus horseradish peroxidase (HRP) kit (DAKO, UK). First, an endogenous peroxidase supplied with the kit was applied for 5 min at room temperature. The samples were then washed, incubated in 10% casein at room temperature for 15 min and then washed again. A 1 : 100 (10 *μ*g ml^−1^ protein) concentration of affinity-pure rabbit anti-human Glut-1 (Alpha Diagnostic International, TX, USA) was applied and the sections were incubated for 1 h at 37^o^C. A rabbit IgG fraction (DAKO) at an identical protein concentration was used as a negative control. Following washing, the secondary antibody (goat anti-rabbit/HRP) was applied to the sections for 30 min at room temperature. A substrate–chromogen solution containing 3.3′-diaminobenzidine in a buffered substrate solution, supplied in the kit, was applied at room temperature for 7 min. After rinsing in water, the slides were counterstained with Gills haematoxylin, dehydrated and mounted. The batch to batch variation was excluded by running sections from the same biopsy through more than one batch, and running one biopsy section through all the batches.

### Scoring method

Glucose transporter-1 expression in tumour cells was evaluated using a semiquantitative scoring method: score 0=absence of immunostaining; score 1=1–10% of cells stained; score 2=10–50% of cells stained; and score 3=>50% of cells stained. No account was taken of the intensity of staining, and ulcerated or necrotic areas were excluded from the evaluation. For each tumour section, three areas were scored: a general score for the whole tumour section, a score for the superficial part of the tumour (approximately nearest one-third from the luminal surface) and a score for the deep part (approximately deepest one-third from the luminal surface). Intraobserver reliability was tested by the same pathologist re-evaluating 20 slides chosen at random after a 4-week gap.

### Statistical analysis

Previous studies have analysed Glut-1 expression as either positive (score 1–3) *vs* negative (score 0) or strongly positive (score 3) *vs* the rest (score (0–2) (Younes *et al*, 1997; Haber *et al*, 1998; Airley *et al*, 2001). It was therefore decided to analyse our patients using these two categories. Correlations between tumour characteristics and the expression of Glut-1 were obtained using a two-tailed Spearman's rank correlation and Fisher's exact test. Survival analysis was by the Kaplan–Meier method, and prognostic factors were assessed by log-rank statistics. Analyses were carried out for overall, metastasis-free and local recurrence-free survival. Bivariate analyses were used to test for the independence from other potential prognostic factors including T stage, nodal stage and tumour size.

## RESULTS

### Expression of GLUT-1

**Figure 1:**
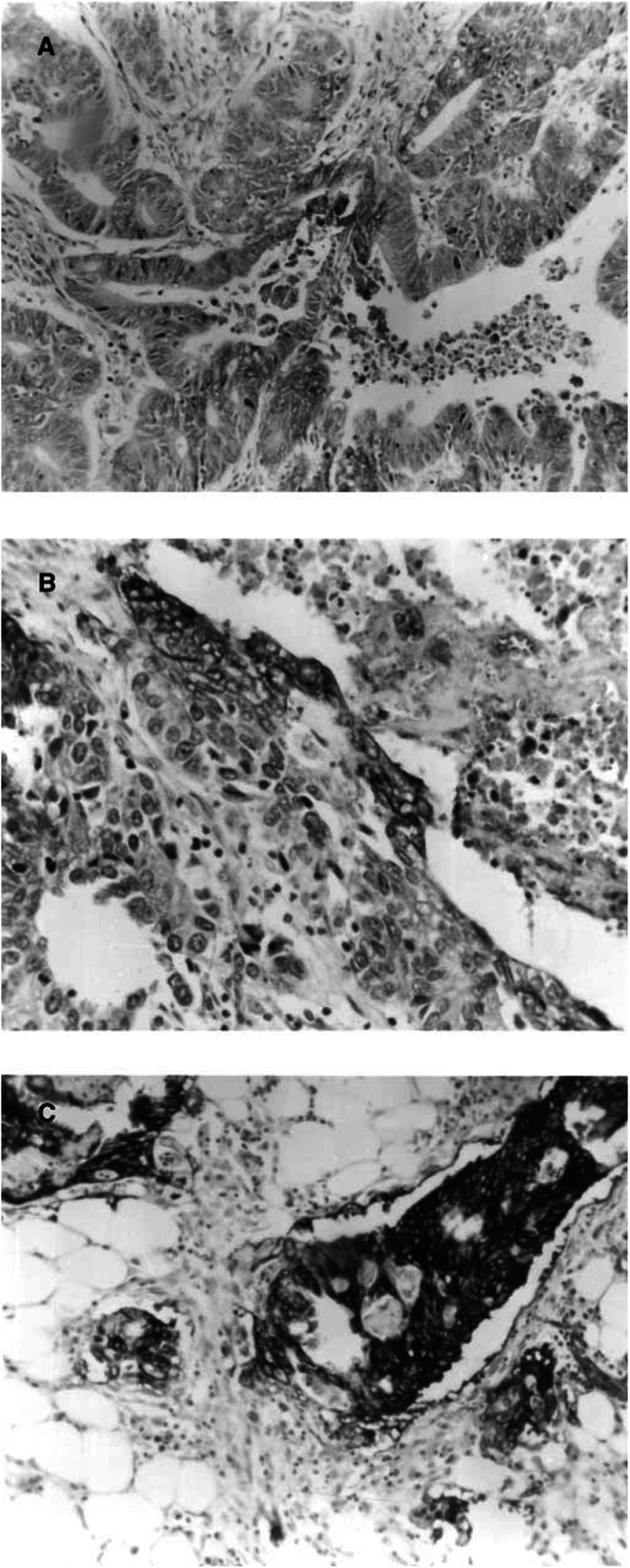
(**A**) Score 1. Less than 10% of the cells are stained by anti-Glut-1 (magnification × 10). (**B**) Score 2. Between 10 and 50% of the cells are stained with Glut-1. Strong membranous staining is seen adjacent to an area of necosis. (magnification × 20). (**C**) Score 3. More than 50% of cells are stained with Glut-1 at the invasive border. (magnification × 20)

Glucose transporter-1 expression was only observed as membranous staining in tumour cells. Erythrocytes within blood vessels were also seen to stain strongly for Glut-1, which served as an internal control. Perinecrotic and periulcerative areas stained strongly. Two patterns of staining were observed, a focal pattern and a more diffuse staining. Although staining was increased around necrotic areas, the patchy staining pattern was not consistently localised to necrosis. In addition, more cells were positive in the deeper portion of the tumour compared to the superfical luminal part. Normal cells and tumour stroma did not stain for Glut-1. There was a significant correlation between the first and the second score for the general (*r*=0.87, *P*<0.0001), superficial (*r*=0.59 *P*=0.03) and deep (*r*=0.82, *P*<0.0001) parts of the tumour. Figure 1

illustrates sections that were scored 1,2 and 3.

### Distribution of patients according to GLUT-1 expression

Of the 43 cases examined, 70% expressed Glut-1. The distribution of scores is shown in Table 2

**Table 2:** Distribution of scores

**Score**	**0**	**1**	**2**	**3**
Patients (number)	30% (13)	42% (18)	21% (9)	7% (3)

. There was a strong correlation between the overall tumour score and the scores for the deep (*r*=0.91, *P*<0.0005) and superficial (*r*=0.89, *P*<0.0005) parts of the tumour.

### Correlation of GLUT-1 expression in relation to clinical factors

The distribution of Glut-1 expression according to Duke's stage and nodal status is shown in Table 3

**Table 3:** Distribution of scores by Duke's stage and nodal status

**Score**	**0**	**1**	**2**	**3**
Stage B	7	11	8	1
Stage C	6	7	1	2
Node negative	8	10	8	1
Node positive	5	8	1	2

. There was no correlation between the degree of Glut-1 immunostaining and tumour stage (*r*=−0.047, *P*=0.77). The patients were also grouped according to the presence or absence of nodal metastasis; however, there was no significant difference in the metastasis status of patients whose tumours showed negative (score 0) or positive (scores 1–3) Glut-1 staining (*P*=0.52, Fisher's exact test). Likewise, there was no significant difference in the metastasis status of patients whose tumours had weak (score 0–2) or heavy (score 3) Glut-1 staining (*P*=0.30, Fisher's exact test). Furthermore, there was no significant association between Glut-1 expression and either tumour grade (*r*=0.10, *P*=0.58) or pathological size (*r*=−0.02, *P*=0.89).

### Relationship between Glut-1 expression and outcome

To examine whether the group of patients studied had similar characteristics to larger groups of patients with rectal cancer, we analysed the relationship between treatment outcome and established clinical prognostic factors (tumour stage, nodal status and tumour size). As expected, advanced stage was a significant adverse prognostic factor (*P*=0.023). In addition, the 5-year actuarial survival was higher for patients with node negative (89%) *vs* positive (56%) tumours (*P*=0.009), and tumours that were pathologically <5 cm (89%) rather than ⩾5 cm (67%) in diameter (*P*=0.06). Grade was a borderline significant prognostic factor for survival (*P*=0.06).

**Figure 2:**
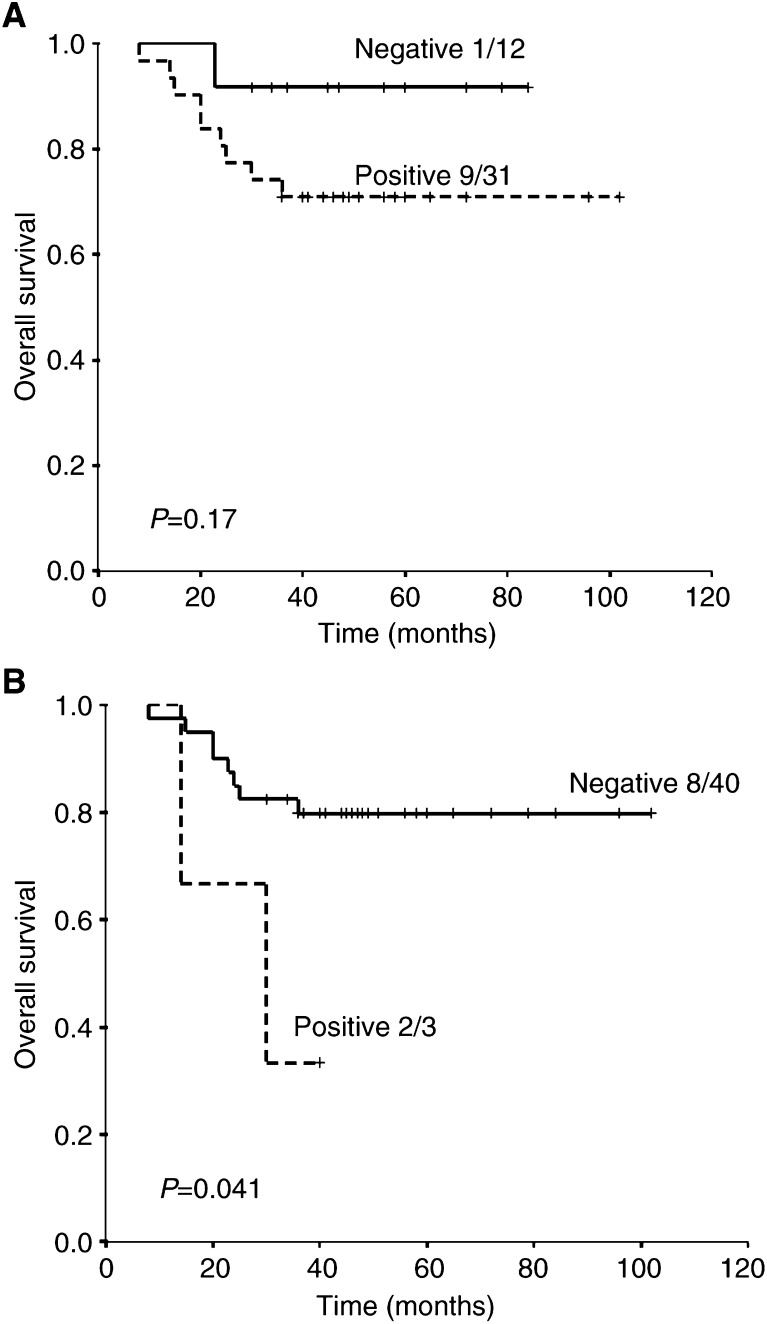
Overall survival for (**A**) score 0 (negative) *vs* 1–3 (positive) and (**B**) score 0–2 (negative) *vs* 3 (positive).

There was no significant difference in the overall survival of patients with Glut-1 positive (score 1–3) *vs* negative (score 0) tumours (71 *vs* 92% at 5 years; *P*=0.17). There was, however, a significant difference in overall survival for patients with strongly positive tumours (score 3) compared to those with a score of 0–2 (33% *vs* 80%, respectively; *P*=0.041). Survival curves are shown in Figure 2

**Figure 3:**
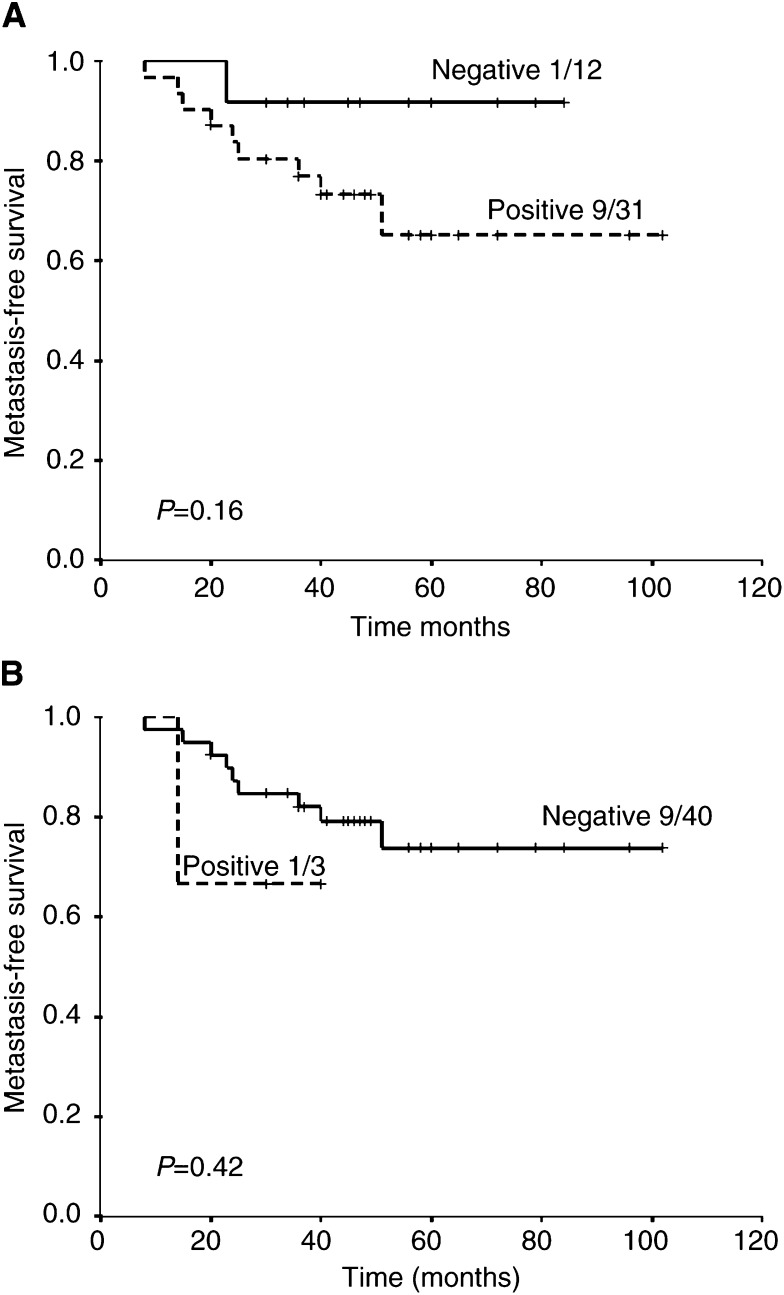
Metastasis-free survival for (**A**) score 0 (negative) *vs* 1–3 (positive) and (**B**) score 0–2 (negative) *vs* 3 (positive).

**Figure 4:**
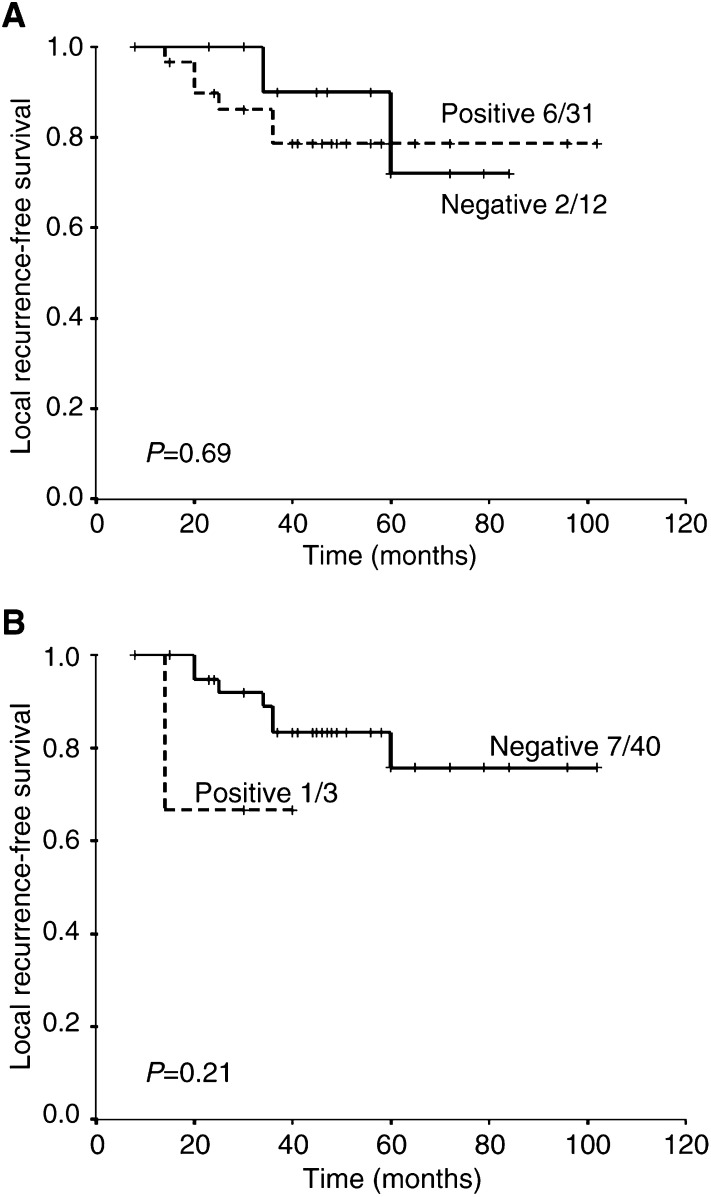
Local recurrence-free survival for (**A**) score 0 (negative) *vs* 1–3 (positive) and (**B**) score 0–2 (negative) *vs* 3 (positive).

. Strong Glut-1 expression in tumours appeared to be associated with a poor metastasis-free survival (Figure 3

) rather than local control (Figure 4

).

Bivariate analyses were performed to test for the independence from tumour stage, nodal status and tumour size. Glucose transporter-1 expression remained a significant prognostic factor for overall survival after allowing for tumour stage (*P*=0.0052) and tumour size (*P*=0.05), but not nodal status (*P*=0.16).

### Glut-1 expression in the superficial *vs* deep part of the tumour

Scores from the deep part of the tumour were also prognostic for overall survival (82 *vs* 25% actuarial survival at 5 years for score 0–2 *vs* score 3, respectively; *P*=0.003). However, the Glut-1 expression of the superficial part of the tumour was not prognostic for survival (actuarial survival at 5 years 78 *vs* 50%, *P*=0.46 for score 0–2 *vs* score 3, respectively).

### Independence of Glut-1 staining to known prognostic factors

In order to assess the degree of Glut-1 staining as an independent prognostic marker, all factors that had been significant for overall survival on log-rank univariate analysis (tumour stage, nodal status, strongly positive *vs* the rest for the whole tumour section and the deep part of the tumour) were included in a multivariate Cox regression analysis. The only factor that was prognostic for survival was the presence of Glut-1 staining in the deep portion of the tumour (*P*=0.013).

## DISCUSSION

The presence of hypoxia in tumours has been shown to correlate with resistance to therapy and reduced survival. Therefore, pretreatment assessment of tumour hypoxia might enable prediction of patients likely to have a poor outcome, and who would be suitable for hypoxia-modifying treatment. Polarographic needle electrodes have been used to give a quantitative, pretreatment measurement of tumour hypoxia that correlates with outcome for patients with carcinoma of the cervix (Hockel *et al*, 1996; Fyles *et al*, 1998), head and neck (Nordsmark *et al*, 1996; Brizel *et al*, 1997) and soft tissue sarcoma (Brizel *et al*, 1996). However, the method is invasive and limited to accessible tumours greater than 2 cm in diameter. Furthermore, any measurement will also include areas of necrosis that can bias the results to falsely low values. An alternative method is the immunohistochemical assessment of bound nitroimadazoles, such as pimonidazole, injected prior to biopsy (Kennedy *et al*, 1997; Nordsmark *et al*, 2001). This requires an added intervention and, as yet, there are only limited data on their relationship with treatment outcome. This has prompted a number of groups to examine potential endogenous markers of hypoxia, which include proteins that are upregulated under hypoxia. The transcription factor HIF-1*α* has been the most widely studied potential endogenous marker of hypoxia (Aebersold *et al*, 2001; Haugland *et al*, 2002; Janssen *et al*, 2002; Koukourakis *et al*, 2002), along with its downstream proteins such as carbonic anhydrase IX (CAIX) (Giatromanolaki *et al*, 2001; Koukourakis *et al*, 2001; Loncaster *et al*, 2001), vascular endothelial growth factor (Loncaster *et al*, 2000; Koukourakis *et al*, 2002) and Glut-1 (Airley *et al*, 2001, 2003). The advantage of using intrinsic markers of hypoxia is that the approach is simple and quick, and could be potentially applied to a wide variety of solid tumour types.

In the present study, we examined the pattern and extent of Glut-1 expression in adenocarcinoma of the rectum as a potential endogenous marker of hypoxia. A total of 70% of tumours expressed Glut-1 to a varying degree. Glucose transporter-1 expression was seen to localise around areas of necrosis and ulceration, while little staining was seen in stromal or normal tissue. In general, two patterns of staining were observed, diffuse and patchy, the latter showing both colocalisation with necrosis and non-necrotic areas. Other studies have reported similar patterns of staining (Haber *et al*, 1998; Airley *et al*, 2001). In addition, two recent studies have shown that the levels of Glut-1 expression in carcinoma of the cervix correlates with the level of tumour hypoxia measured using either polarographic needle electrodes (Airley *et al*, 2001) or pimonidazole staining (Airley *et al*, 2002). In the former study, the strongest correlation was seen with the most hypoxic fraction of polarographic needle electrode measurements, leading to the suggestion that Glut-1 scores might represent the level of chronic or diffusion-limited hypoxia, while in the second study, there was a good, but not exact, colocalisation of pimonidazole staining and Glut-1 expression. Taken together, these findings suggest a link between hypoxia and Glut-1 expression in human carcinoma of the cervix. However, several factors could reduce the usefulness of Glut-1 as a hypoxic marker and explain the diffuse pattern of staining observed in some of the tumours. Glucose transporter-1 expression is known to be stimulated by a number of other stimuli including growth factors, thyroid hormone, alkaline pH and oncogenic transformation (Flier *et al*, 1987; Hakimian *et al*, 1991; Kuruvilla *et al*, 1991). Furthermore, it is dually controlled by both hypoxia and reduced oxidative phosphorylation in the absence of hypoxia (Behrooz *et al*, 1997). Chronic hypoxi**a** leads to increased production of Glut-1 that is mediated via the transcription factor HIF-1. Although it was previously thought that HIF-1 was only stabilised under hypoxia, it is now known to be constitutively expressed in some tumours due to genetic alterations occurring during malignant transformation (Jiang *et al*, 1997; Feldser *et al*, 1999; Zhong *et al*, 2000). This might therefore explain the diffuse pattern of staining and the patchy noncolocalisation with necrosis observed in some of the tumours in the present study.

In order to exploit the measurement of Glut-1 expression as a marker of hypoxia in different types of tumours, there is a need to demonstrate that the marker can provide prognostic information in each tumour type of interest. Previous studies have shown a relationship between Glut-1 expression and overall survival in colon cancer (Haber *et al*, 1998) and lung cancer (Younes *et al*, 1997), and disease-free survival in ovarian cancer (Cantuaria *et al*, 2001). In addition, a significant association between Glut-1 expression and metastasis-free survival, but not disease-free survival or local recurrence, has been reported in patients with cervical carcinoma (Airley *et al*, 2001). In the present study, we were able to show in rectal cancers that strong expression of Glut-1 (>50% of tumour cells) was a significant prognostic factor for overall survival independent of tumour stage and tumour size, and on multivariate analysis the presence of strong staining in the deep part of the tumour was the only significant factor for overall survival. We also observed a nonsignificant relationship with metastases-free survival, but not local recurrence-free survival, for the presence of staining (1–3) *vs* non (0). However, larger numbers of patients would be required to confirm this.

It is of interest that in the study of Airley *et al* (2001), where Glut-1 expression was prognostic for metastasis-free survival, but not overall or local recurrence-free survival, patients with cervical carcinoma were treated with radiotherapy. In our study, patients were predominantly treated with surgery, and Glut-1 was predictive for overall and metastasis-free survival but again not local recurrence-free survival. It might be that Glut-1 expression reflects more severe and longer duration hypoxia, especially as in our study intense staining (score 3) showed the best correlation with outcome and heavy staining is more indicative of *de novo* Glut-1 synthesis which only occurs after prolonged hypoxia (Shetty *et al*, 1992; Behrooz *et al*, 1997). This in turn might be more reflective of the tumours propensity to form distant metastases, associated with hypoxia rather than resistance to radiotherapy and is consistent with previous experimental and clinical data (Fyles *et al*, 2002).

We found no significant relationship between the expression of Glut-1 in tumours and the accepted clinical prognostic factors of tumour stage, depth of invasion (T stage), nodal status, tumour size and grade of differentiation. In contrast, a previous study in patients with mainly colon cancer showed that extensive staining (>50% of cells) for Glut-1 correlated significantly with the presence of nodal metastases (Haber *et al*, 1998). Similarly, no consistent picture has emerged for any relationships between clinical prognostic factors and the level of tumour hypoxia measured using polarographic needle electrodes (Hockel *et al*, 1996; Nordsmark *et al*, 1996; Pitson *et al*, 2001). The fact that no correlation was found in our study might reflect the low number of patients included and**/**or the inhomogeneous treatment given. However, the lack of association and the fact that only strong staining in the deep part of the tumour was a significant factor for overall survival on Cox regression analysis suggests that Glut-1 expression in rectal tumours might give additional information over and above that provided by the established clinical prognostic factors. Studies on a larger group of patients are required to clarify this issue.

Of interest in our study was the finding that more cells were seen to express Glut-1 in the deeper compared to the superficial (corresponding with the luminal surface) parts of tumours. In addition, no relationship was found between patient outcome and Glut-1 expression in the superficial part of the tumours. The latter observation is difficult to interpret because of the lack of published data on the oxygenation status of rectal tumours. However, the data do highlight the potential problem of intratumour heterogeneity and the need to take multiple biopsies from different parts of rectal tumours. Taking a mean of three biopsies per patient, as used in our study, is recommended for other studies in this area.

In the results presented here, Glut-1 was expressed in two-thirds of the rectal tumours examined, and high expression was associated with a poor treatment outcome. In addition to Glut-1, several potential endogenous markers of hypoxia are currently under investigation including HIF-1*α*, HIF-2*α*, CAIX and VEGF. Studies suggest that different levels and/or durations of tumour hypoxia are required to upregulate each protein (Giatromanolaki *et al*, 2001; Olive *et al*, 2001). It is possible, therefore, that a combination of markers might be required to fully evaluate hypoxia within a given tumour type. Furthermore, several studies have shown that combining protein expression with other factors, such as the level of vascularity, improves the definition of patient groups with differing prognoses (Koukourakis *et al*, 2001,^2002^). This is the first study of Glut-1 expression in purely rectal carcinoma. Rectal cancer is an interesting area as preoperative radiotherapy or chemoradiotherapy is being increasingly used as part of the treatment. Furthermore, there is still debate as to which node-negative patients should receive adjuvant chemotherapy. As several different regimens are being used, a better understanding of the individual tumour microenvironment might enable a more rational application of these therapies along with the incorporation of hypoxia-modifying treatments for those patients with hypoxic tumours.
